# Pied varus équin de l’adulte traité par arthrodèse tibiocalcanéenne: à propos de deux cas (3 pieds)

**DOI:** 10.11604/pamj.2018.30.222.15297

**Published:** 2018-07-20

**Authors:** Mohamed Mahmoud El Hacen, Yacoub Mohamed Sghair, Noura Biha, Abdoulay Aw, Moez Trigui, Chekh Né

**Affiliations:** 1 Orthopedic Department, Nouakchott Military Hospital, Faculty of Medicine of Nouakchott, Mauritania; 2 Pediatric Surgery, CHME-Nouakchott, Faculty of Medicine of Nouakchott, Mauritania; 3 Rheumatology Department, Nouakchott Military Hospital, Faculty of Medicine of Nouakchott, Mauritania; 4 CTGB-CHN, Nouakchott, Mauritania; 5 CHU-Habib Bourguiba de Sfax, Faculty of Medicine of Sfax-Tunisia; 6 CTGB-CHN/Faculty of Medicine of Nouakchott, Mauritania

**Keywords:** Pied bot varus équin, adulte, arthrodèse tibiocalcanéenne, Talipes equinovarus, adult, tibiocalcaneal arthrodesis

## Abstract

Le pied bot varus équin (PBVE) invétéré est une attitude vicieuse du pied qui ne repose plus sur le sol par ses points d'appui normaux mais par son bord externe et sa pointe. Lorsque cette attitude vicieuse persiste longtemps, elle évolue vers des déformations ostéoarticulaires importantes à l'âge adulte. Le PBVE fixé constitue une bonne indication d'arthrodèse tibio-calcanéenne. Celle-ci permet dans la majorité des cas d'obtenir un appui plantigrade indolore et stable. Le but de ce travail est d'évaluer les résultats cliniques et radiologiques de l'astragalectomie avec adhèse tibiocalcanéenne. Nous rapportons une série de 3 cas d'arthrodèse tibio-calcanéenne pour déformation grave de l'arrière-pied associant un varus et un équin important et gênant chez l'adulte. Les résultats étaient jugés satisfaisants avec un appui plantigrade indolore et stable. Avec fusion de l'arthrodèse à 90 jours, sans inégalité pour le cas bilatéral et un raccourcissement de 2cm compensé par une semelle à droite pour le cas unilatéral. Dans les déformations importantes et fixées de la cheville et du PBVE, l'arthrodèse tibio-calcanéenne permet dans la majorité des cas d'obtenir un appui plantigrade indolore et stable sans risque vasculaire, ni cutané.

## Abstract (en)

Inveterate clubfoot (inveterate talipes equinovarus, ITEV) is a vicious attitude of the foot which doesn’t rest on the floor through its normal base of support but weight is thrown to the outer side of the foot and to the foot point. When this vicious attitude persists a long time, it results in severe osteoarticular deformities in adults. Fixed ITEV is a good indication for tibiocalcaneal arthrodesis. In the majority of cases this provides painless and stable plantigrade foot. This study aims to assess the clinical and radiological outcomes of astragalectomy with tibiocalcaneal fusion. This case series involves 2 adult patients undergoing tibiocalcaneal arthrodesis due to severe and annoying equinovarus rearfoot deformity. Outcome was satisfactory with painless and stable plantigrade foot. At 90-day follow-up the patient with bilateral clubfoot had no inequality while the patient with unilateral clubfoot had a shortening of 2 cm compensated by right sole. In the majority of cases with severe and fixed deformities of the ankle and of the ITEV, tibiocalcaneal arthrodesis allows for painless and stable plantigrade foot, without vascular or cutaneous risk.

## Introduction

Le pied bot varus équin (PBVE) (invétéré) est une attitude vicieuse du pied qui ne repose plus sur le sol par ses points d'appui normaux mais par son bord externe (varus) et sa pointe (équinisme). Lorsque cette attitude vicieuse persiste longtemps [invétéré], elle évolue vers des déformations ostéoarticulaires importantes à l'âge adulte. L'astragalectomie est utilisée dans les déformations majeures fixées du pied particulièrement en varus équin. Qu'il s'agît d'une déformation congénitale, multiopérée ou négligée, d'une séquelle d'un pied neurologique ou d'une séquelle traumatique, l'objectif est d'obtenir un appui plantigrade, un pied indolore et fonctionnel. Le but de ce travail est d'évaluer les résultats cliniques et radiologiques de l'astragalectomie avec adhèse tibiocalcanéenne. Différentes techniques ont été proposées pour traiter un PBVE chez l'adulte. L'importance de la déformation fixée exige pour la correction une résection osseuse à un niveau ou à un autre. Johnson et al. [1] ont proposé la pan-arthrodèse entraînant un blocage définitif de l'arrière-pied et du médio-pied. Hall et Calvert [2] ont rapporté une série d'arthrodèses associées à l'artifice d'arthrolyse de Lambrinudi, qui consiste en une ostéotomie triangulaire de soustraction au niveau de la sous-talienne, permettant de corriger l'équin. On peut aussi réaliser une correction progressive du pied varus en recourant à la fixation externe selon Ilizarov. Nous rapportons une série rétrospective de 3 cas d'arthrodèse tibio-calcanéenne pour déformation grave de l'arrière-pied associant un varus et un équin important.

## Patient et observation

**Figure 1:**
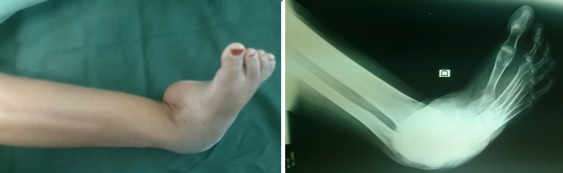
Aspect clinique initial + radiographie initiale

**Figure 2:**
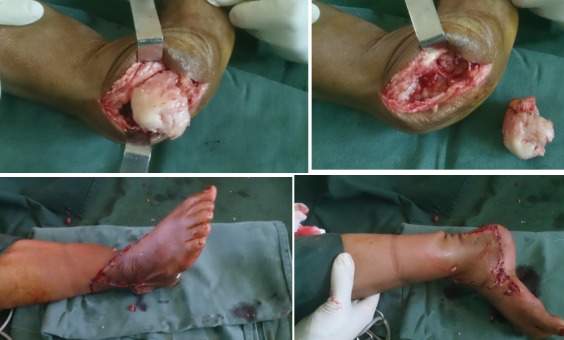
Astragalectomie

**Figure 3:**
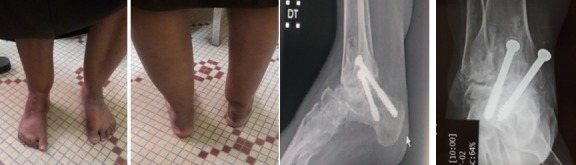
Aspect clinique + radiographie à 3 ans PO

**Premier cas:** madame L.F âgée de 43ans qui a consulté pour un pied bot varus équin invétéré droit, elle n'a jamais eu un traitement auparavant, elle présentait une gêne douloureuse à la marche avec boiterie et lombalgie chronique en rapport avec l'inégalité des membres inférieurs. Elle marchait sur le dos du pied droit. A l'examen clinique nous avons noté une amyotrophie manifeste de tous les muscles de la jambe droite. Avec un pied bot varus équin droit irréductible (Figure 1). L'opération avait pour but de corriger l'ensemble des déformations des pieds pour restaurer un appui plantigrade, supprimer la douleur, faciliter le chaussage avec des semelles pour compenser l'inégalité des membres inférieurs afin de réduire le retentissement sur le bassin et le rachis lombaire et redonner à la patiente un aspect esthétique acceptable des pieds. Un examen radiologique (Figure 1) et un bilan biologique préopératoire ont été réalisés. Elle a été opérée sous anesthésie locorégionnale, avec un garrot à la racine du membre, la voie d'abord a été antéro-latérale commençant à 6 travers de doigts au-dessus de la pointe de la fibula et se terminant à 4 travers de doigt en dessous. L'astragalectomie a été réalisé pour les deux pieds, La malléole externe a été réséquée partiellement car elle empêchait tout contact tibio-calcanéen., une ténotomie avec allongement du tendon d'Achille a été effectuée. L'avivement de l'extrémité inférieure du tibia et du calcaneum, a été fait au ciseau a os; pince gouge et à la curette (Figure 2). La fixation a été réalisée par des vis tibiocalcanéennes avec mise en place d'une botte plâtrée pendant 45 jours. Les résultats étaient jugés très bon au recul de 03 ans avec appui plantigrade du pied avec un pied creux résiduel indolore et à la radiographie une fusion de l'arthrodèse (Figure 3) avec inégalité de 02cm des membres inférieurs compensé par une semelle à droite.

**Figure 4:**
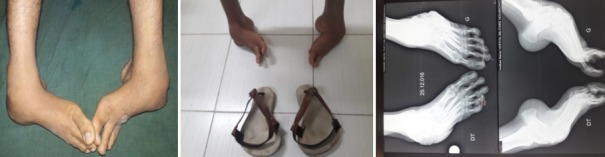
Aspect clinique initial + chaussure + aspect radiologique initial

**Figure 5:**
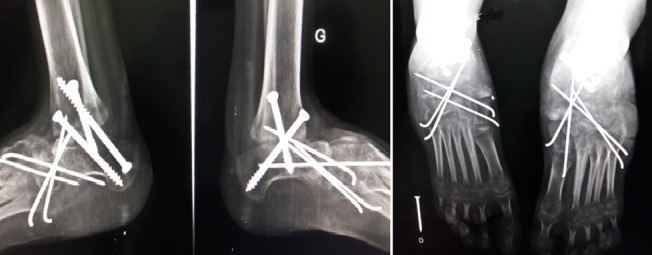
Aspect radiologique à J45 postopératoire

**Figure 6:**
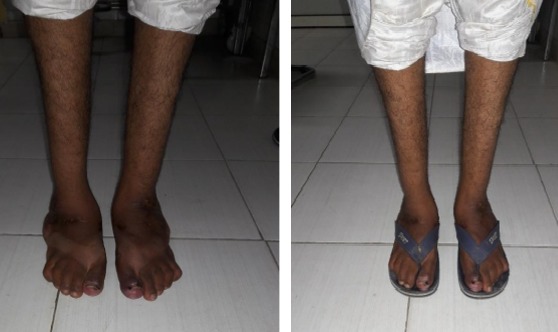
Aspect clinique a 10 mois postopératoire

**Figure 7:**
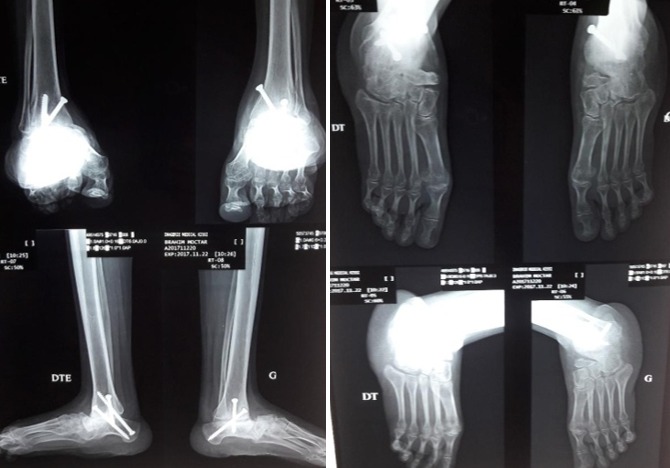
aspect radiologique à 10 mois postopératoire

**2^ème^ cas:** monsieur B.M âgé de 23 ans qui a consulté pour déformations sévères des pieds avec pied bot varus équin invétéré bilatérales, il n'a jamais eu un traitement auparavant. Il présentait une gêne douloureuse à la marche et des difficultés de chaussage avec surtout un aspect esthétique gênant. Il marchait sur les dos des pieds avec orthèse artificielle comme chaussure (Figure 4). A l'examen clinique nous avons noté une amyotrophie manifeste de tous les muscles de deux jambes. Il s'agissait de pieds bots varus équins irréductibles. L'opération avait pour but de corriger l'ensemble des déformations des pieds pour restaurer un appui plantigrade, supprimer la douleur, faciliter le chaussage et redonner au patient un aspect esthétique acceptable des pieds. Un examen radiologique (Figure 5) et un bilan biologique préopératoire a été réalisés. Il a été opéré sur les deux pieds en même temps sous anesthésie générale, avec un garrot à la racine du membre, la voie d'abord a été antéro-latérale commençant à 6 travers de doigts au-dessus de la pointe de la fibula et se terminant à 4 travers de doigt en dessous. L'astragalectomie a été réalisée pour les deux pieds, la malléole externe a été réséquée partiellement car elle empêchait tout contact tibio-calcanéen, une ténotomie avec allongement du tendon d'Achille a été effectuée. L'avivement de l'extrémité inférieure du tibia et du calcanéum a été fait à l'aide de ciseau à os; pince gouge et à la curette. Au niveau de l'avant pied nous avons réalisé un avivement avec arthrodèse medio-tarsienne et une plastie des extenseurs des orteils. La fixation a été réalisée par des vis tibiocalcanéennes et des embrochages des pieds avec mise en place d'une botte plâtrée pendant 45 jours. Les suites opératoires ont été favorables et les résultats ont été appréciés en fonction de l'aspect morphologique, fonctionnel, et radiologique du pied et ils étaient jugés très bon avec appuis plantigrade du pied sans déformation résiduelle et indolore (Figure 6) et à la radiographie. La consolidation a été acquise en 90 Jours (Figure 7) et surtout sans inégalité des membres.

## Discussion

Dans les déformations importantes et irréductibles. L'astragalectomie permet d'obtenir un appui plantaire indolore et stable sans risque vasculaire. Mais elle ne corrige que partiellement l'avant-pied. Dans le deuxième cas des gestes complémentaires au niveau du médio-pied et de l'avant-pied étaient nécessaires. Ceci était confirmé par Johnson et al. [1] qui avaient également insisté sur la difficulté d'obtenir un bon contact entre l'extrémité inférieure du tibia et le calcanéus. Cette intervention entraîne en effet une perturbation importante des rapports anatomiques. Une résection fibulaire distale est parfois nécessaire afin d'obtenir un contact. Itokazu et al. [3] notent que l'astragalectomie n'entraîne qu'un raccourcissement moyen de 2 cm. Ce qui a été confirmé dans notre 1^er^ cas où la déformation est unilatérale avec un raccourcissement de 2 cm. La fusion de l'arthrodèse a été obtenue à 90 jours pour les deux cas. Alvarez et al. [4] ont obtenu une fusion dans tous les cas. Selon Marwen Jlailia et al [5], Les déformations majeures sont source de gêne importante, de conflits rendant le chaussage impossible et peuvent conduire à des ulcérations. Les données de la littérature sont concordantes pour dire que l'astragalectomie est une indication de sauvetage du membre. Elle permet d'obtenir un pied plantigrade et indolore. La fusion de l'arthrodèse n'est pas indispensable à la réussite de l'opération [beaucoup de pseudarthroses sont bien tolérées]. Le but premier de cette chirurgie devrait être l'alignement de l'arrière pied afin de corriger la déformation. Certains ont proposé une correction progressive du PBVE par fixateur externe d'Ilizarov non suivie d'arthrodèse, qui a l'avantage de ne pas nécessiter d'astragalectomie. L'utilisation du fixateur externe d'Ilizarov reste exceptionnelle. Ce fixateur, d'une grande puissance, permet de corriger les déformations historiques des pieds non traités. Cette technique permet de venir à bout de déformations importantes mais augmente considérablement la raideur articulaire même sans abord chirurgical. Les auteurs constatent un maintien des résultats dans le temps et des résultats identiques à ceux de l'arthrodèse mais sans raccourcissement du pied [2cm pour le 1^er^ cas] et sans blocage articulaire. C'est une technique extrêmement séduisante mais complexe dans sa réalisation, douloureuse et longue. Cependant la récidive est fréquente au cours du traitement par fixateur externe d'Illizarov surtout avec les pieds neurologiques [6]. Nous pensons qu'il faut faire une arthrodèse une fois obtenue la correction par Ilizarov. Nous restons fidèles à l'arthrodèse tibio-calcanéenne avec résection de l'astragale.

Nous n'avons pas enregistré de cas de pseudarthrose. Cependant selon ASSENCIO G. [7] le taux de pseudarthrose varierait de 0 à 36% selon les séries au cours de la double arthrodèse. Nos résultats sont nettement encourageants par rapport à ceux obtenus par RIBAULT L. [8] qui ont obtenu 13 résultats satisfaisants contre 6 résultats non satisfaisants sur un total de 19 pieds bots varus équins du grand enfant, de l'adolescent et de l'adulte traités par libération postéro-interne associée secondairement à un temps osseux qui n'a été envisagé qu'au moins 3 mois après. La différence entre cet auteur et nous est que, nous avons fait en un seul temps opératoire tous les gestes nécessaires à la correction parfaite du PBVEI. La qualité de nos résultats s'explique par le fait que l'association de ces deux techniques dans le même temps opératoire nous a permis une correction plus facile des déformations du pied et un contrôle à vue de la résection osseuse qu'on peut continuer à faire à la demande. Selon Karima Atarraf et al [6]. L'arthrodèse sous-talienne et médio tarsienne est indiquée dans le traitement du PBVEI comme un moyen de sauvetage permettant une correction des déformations et procurant ainsi des résultats meilleurs. SERINGE R [9] à propos de la double arthrodèse tardive pour pied bot a écrit ceci « la réalisation correcte d'une double arthrodèse ne saurait se faire sans utiliser deux voies d'abord distinctes: l'une externe habituelle à toute double arthrodèse, l'autre interne permettant une libération interne et un allongement du jambier postérieur ».

## Conclusion

Dans les déformations importantes et fixées de la cheville et du pied en varus équin, l'arthrodèse tibio-calcanéenne permet dans la majorité des cas d'obtenir un appui plantigrade indolore et stable sans risque vasculaire, ni cutané.

## Conflits d’intérêts

Les auteurs ne déclarent aucun conflit d'intérêts.
